# Value-based care in obstetrics: comparison between vaginal birth and caesarean section

**DOI:** 10.1186/s12884-021-03798-2

**Published:** 2021-04-26

**Authors:** Romulo Negrini, Raquel Domingues da Silva Ferreira, Daniela Zaros Guimarães

**Affiliations:** Maternal and Child Department, Hospital Israelita Albert Einstein, Av. Albert Einstein, 627 - Jardim Leonor, São Paulo, SP 05652-900 Brazil

**Keywords:** Healthcare cost, Obstetrics, Delivery of healthcare, Quality of healthcare, Birth setting, Obstetric delivery, Cesarean section

## Abstract

**Background:**

Healthcare costs have substantially increased in recent years, threatening the population health. Obstetric care is a significant contributor to this scenario since it represents 20% of healthcare. The rate of cesarean sections (C-sections) has escalated worldwide. Evidence shows that cesarean delivery is not only more expensive, but it is also linked to poorer maternal and neonatal outcomes. This study assesses which type of delivery is associated with a higher healthcare value in low-risk pregnancies.

**Results:**

A total of 9345 deliveries were analyzed. The C-section group had significantly worse rates of breastfeeding in the first hour after delivery (92.57% vs 88.43%, *p* < 0.001), a higher rate of intensive unit care (ICU) admission both for the mother and the newborn (0.8% vs 0.3%, *p* = 0.001; 6.7% vs 4.5%, *p* = 0.0078 respectively), and a higher average cost of hospitalization (BRL14,342.04 vs BRL12,230.03 considering mothers and babies).

**Conclusion:**

Cesarean deliveries in low-risk pregnancies were associated with a lower value delivery because in addition to being more expensive, they had worse perinatal outcomes.

## Introduction

Healthcare costs are a growing concern. They have rapidly risen and constitute a threatening to health access throughout the world. To discuss how to solve this problem is essential by also reviewing the financing model of the various fields of healthcare.

According to a report by the World Health Organization (WHO), health expenditure is growing more rapidly than the global economy and represents 10% of the global Gross Domestic Product (GDP) [1].

In Brazil, there is a private and a public healthcare system, the latter is called the Unified Health System (SUS). Between 2010 and 2017 the ratio of health spending to GDP has grew from 8% to 9,2% [2]. The majority of this value is paid by households in the private sector. While government expenditure decreased from 5,3% of the GDP in 2010 to 3,9% in 2017, household disbursement increased from 4,3% to 5,3% for the same period [3]. This context generated a transition of more than 3 million users of the supplementary health system to the SUS between 2015 and 2017, according to data from the National Association of Private Hospitals (ANHAP) [4].

The rise in health costs affects all spheres of care; however, the maternal-perinatal represent a large share of these expenditures. In Brazil, maternal and neonatal hospitalizations represented almost 12% of all hospital admissions in 2020, whereas in 2019 they accounted for 9,4% [5]. In the United States, according to the *Agency for Healthcare Research and Quality*, maternal and neonatal hospitalizations represent more than 20% of all hospital admissions. They also represent the largest isolated category of hospital expenses, reaching more than a quarter of the amount transferred from health insurance companies [6, 7]. Besides that, the costs related to maternal and neonatal care grew about 90% between 2003 and 2013, they reached more than 127 billion dollars annually in that country [8, 9].

Many of these results are due to the increase of C-section, which seems to have poorer maternal and fetal clinical outcomes and higher costs [10–13].

Cesarean deliveries are associated with a higher rate of newborn admissions to neonatal intensive care units (NICU), longer hospital stay, and greater use of human resources for assistance [13]. Also, unnecessary C-sections seem to increase the risk of parturient as their inadvertent practice may increase in 3.7 times the chances of maternal death, and approximately 5 times of those of amniotic embolism, along with being related to a higher future incidence of abnormal placental insertion [11].

For *Medicaid*, the average costs involved in cesarean delivery including prenatal care, childbirth, and postnatal care are US$13,590.00 per event. On the other hand, for vaginal deliveries this cost is about 30% lower, i.e., US$9,131.00 [14].

Despite all these issues mentioned, C-section rates have risen in some countries. Although the ideal and safe rate for cesareans should be around 15–18%, there was an increase from just over 20% in 1996 to almost 33% in 2011 in the United States [7, 15]. In Brazil, the situation is even more critical. Considering both the public and private systems, there was an increase from 40% to nearly 55% in the same period, with the private system accounting for values exceeding 80% [16].

This means that less value has been delivered since poorer outcomes are associated with a higher cost.

To establish what led to the current scenario of indiscriminate use of C-sections is complex, especially in Brazil, but some factors have been listed, such as: judicialization of health (not only due to the demand for access to new technologies, but also due to questioning of unexpected outcomes such as childbirth anoxia), patient’s pain-related fear, lack of training for health professionals in vaginal delivery.

Another contributor to the increase of C-section is the physician-centered care models, which is common in Brazil, mainly in the private sector. These models are associated with a remuneration system that pays equally for vaginal and cesarean deliveries and inadequately privilege dedication to the hours of labor, regardless of the associated complications. Thereby, the prolonged assistance needed in vaginal deliveries ends up making the intervention a convenience one in order to end the birth process without financial losses [17].

These factors explain the lower cesarean rates in the public system, where medical and nursing pay is per shift, compared to the private system, whose medical payment usually occurs per event. Nevertheless, cesarean rates in the public sector are also high due to the other factors mentioned: judicialization of health, patient’s pain-related fear and lack of training for health professionals in vaginal delivery.

In this context, the adoption of a value-based remuneration model is advocated as one of the alternatives to shift this scenario. The Brown’s team defined Value-Based Medicine (VBM) as “the practice of medicine incorporating the highest level of evidence-based data with the patient-perceived value conferred by healthcare interventions for the resources expended” [18]. Consequently, it encompasses three main components: evidence-based decision, patient value-based data and cost-effectiveness in selecting an intervention [19].

Therefore, value may be defined by the quality of the care provided divided by its cost, that is, the clinical result achieved by the amount spent [20]. Clinical result is understood not as the quantity of services to which the patient is submitted, but as the quality of these services in terms of safety and efficiency [21, 22]. This quality needs to be measured objectively, considering processes, which must be based on scientific evidence, or clinical outcomes [19]. This measurement allows a better comparison of the assistance provided by services and professionals.

In summary, VBM is considered to be the search for health deliveries, or interventions, based on scientific and economically sustainable evidence, in which patients perceive benefit, either by greater satisfaction or by reducing complications. This reduction is intrinsically related to satisfaction [19].

Based on the above, the value in health would be greater the better the results measured and the lower the costs.

As value-based medicine clearly involves an economic aspect, it is crucial to establish the ideal remuneration model. To adopt one paying strategy that can encourage the best practices while avoiding waste is advised, as well as considering better outcomes and technically adequate practice [23, 24].

A new question is raised at this point: what exactly better results mean? Since they are related to the values perceived by the patients, which need to be measured, in the same way as costs. Therefore, several measurable factors have been proposed: the percentage of C-section rate; the percentage of births at full-term (over 39 weeks); NICU admission rate; breastfeeding rate; the rate of newborns with an Apgar score less than 7 in the fifth minute of life; maternal readmission rate, among many others [25, 26].

There is still a debate as to whether vaginal deliveries would be associated with higher value delivery in all settings. Consequently, this study proposes to assess which mode of delivery is associated with a greater value delivery in low-risk pregnancies considering clinical results and related costs in a private hospital in Brazil.

## Methods

### Outcomes

Primary outcome: To determine whether C-section or vaginal birth is associated with a greater value delivery in low-risk pregnancies considering clinical results and related costs in a private hospital in Brazil.

Secondary outcome: To compare in both mode of delivery the rate of breastfeeding in the first hour after delivery, the rate of ICU admission both for the mother and the newborn, the average cost of hospitalization, and the hospital readmission up to 30 days after delivery.

### Study design

This study was approved by the ethical committee and conducted in a private hospital. A hospital database (Excel) was used for the analysis, and the data was extracted from the birth record book and validated with information from the electronic medical record. A filter was applied to select the low-risk pregnancies deliveries from this basis, defined as singleton pregnancies at term with cephalic presentation without previous C-section. A retrospective analysis was made from 2016 to 2019.

The average costs per patient (fixed and variable) involved in the overall hospital care of the maternal-fetal binomial were calculated for vaginal and cesarean deliveries considering the period of hospitalization related to that delivery. Costs were calculated based on direct and indirect expenses, which make up tables with extensive items. Direct costs are given by the average value of all items (drugs and materials) posted to the patients’ hospital bill during the hospitalization period. The average indirect costs, on the other hand, are based on the fraction of use of human resources and the contribution to the depreciation of equipment cared for by assistance, according to the time of use and useful life of the equipment. Besides that, the results related to the value delivery for this binomial were compared using the following indicators:

- Breastfeeding rate in the first hour of life;
- NICU admission rate;
- Maternal ICU admission rate.
- Maternal hospital readmission rate within 30 days from delivery;

**Inclusion criteria:**

- Deliveries at a selected hospital from 2016 to 2019;
- Single pregnancies;
- Term pregnancies;
- Cephalic presentation at the time of delivery;
- Absence of previous C-section.

**Exclusion criteria:**

- Pregnancies under 37 weeks;
- Multiple pregnancies;
- Abnormal fetal presentation at the time of delivery;
- Presence of previous cesarean section.

### Statistical analysis

For statistical analysis, the Komogorov-Smirnov test was applied to the database of the variables of interest both for vaginal and for cesarean sections to verify whether the values presented normal distribution, considering the significance of 5% (*p* < 0.05).

For variables in which the values did not present a normal distribution, a non-parametric test for independent samples (Mann-Whitney) was applied. For those with normal distribution, a T-test was applied for independent samples. In all analysis, the significance of 5% (*p* < 0.05) was considered.

## Results

A total of 9345 deliveries were analyzed. Of these 2377 occurred in 2016; 2456 in 2017; 2304 in 2018 and 2208 in 2019. Cesarean rates were 57.1% in 2016, 58.1% in 2017, 55.6% in 2018 and 51% in 2019, as shown in Table 1.

**Table 1:** Total number and percentage of C-sections and vaginal birth per year, including number and percentage of emergent delivery among C-sections

Year	Total deliveries	C-sections (%)		Vaginal Birth (%)
		Total	Emergency	
2016	2377	1357 (57.1)	58 (4.2%)	1020 (42.9)
2017	2456	1425 (58.1)	7 (4%)	1031 (41.9)
2018	2304	1281 (55.6)	62 (4.8%)	1023 (44.4)
2019	2208	1125 (51.0)	63 (5.6%)	1083 (49.0)

The data shows that the groups submitted to C-section and vaginal delivery were very similar in terms of average maternal age, average fetal birth weight and average gestational age at delivery (Table 2).

**Table 2:** Demographic characteristics of the study population stratified by type of delivery and year of occurrence

	C-sections	Vaginal Birth
Year	**Mean Maternal Age (year)**	
2016	33.7 (CI95%, 33.5–33.9)	33.4 (CI95%, 33.2–33.6)
2017	34.0 (CI95%, 33.7–34.2)	33.6 (CI95%, 33.3–33.7)
2018	34.3 (CI95%, 34.0–34.5)	33.9 (CI95%, 33.7–34.1)
2019	34.2 (CI95%, 33.9–34.5)	33.7 (CI95%, 33.6–34.0)
Year	**Average Fetal Weight (grams)**	
2016	3264 (CI95%, 3242–3286)	3256 (CI95%, 3234–3278)
2017	3268 (CI95%, 3246–3290)	3250 (CI95%, 3227–3273)
2018	3273 (CI95%, 3250–3296)	3241 (CI95%, 3216–3266)
2019	3271 (CI95%, 3247–3295)	3236 (CI95%, 3214–3258)
Year	**Average Gestational Age (weeks)**	
2016	39.1 (CI95%, 39.0–39.1)	39.3 (CI95%, 39.1–39.3)
2017	39.1 (CI95%, 39.0–39.2)	39.3 (CI95%, 39.2–39.4)
2018	39.1 (CI95%, 39.0–39.2)	39.3 (CI95%, 39.2–39.4)
2019	39.2 (CI95%, 39.1–39.3)	39.3 (CI95%, 39.2–39.4)

Only the variables “maternal ICU admission” and “readmission up to 30 days from delivery” did not present a normal distribution of values.

**Fig. 1:**
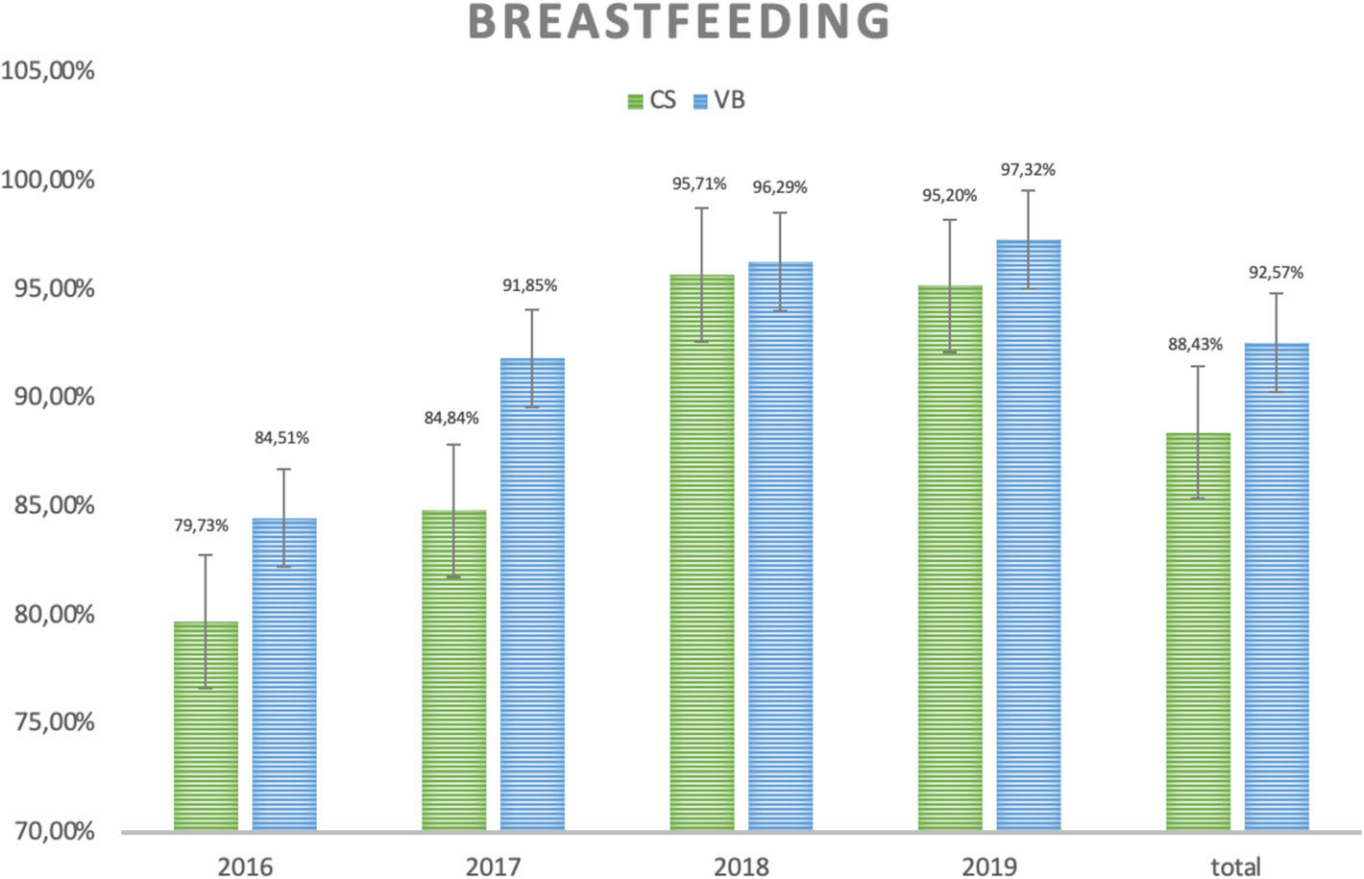
Breastfeeding rate in the first hour of life according to type of delivery and year of occurrence. CS = cesarean sections, VB = vaginal birth

The analysis of breastfeeding rates in the first hour of life shows that although subtly, this practice is more frequent among women who had a vaginal delivery than those who had a C-section (92.57% vs 88.43%, *p* < 0.001), as shown in Fig. 1.

**Fig. 2:**
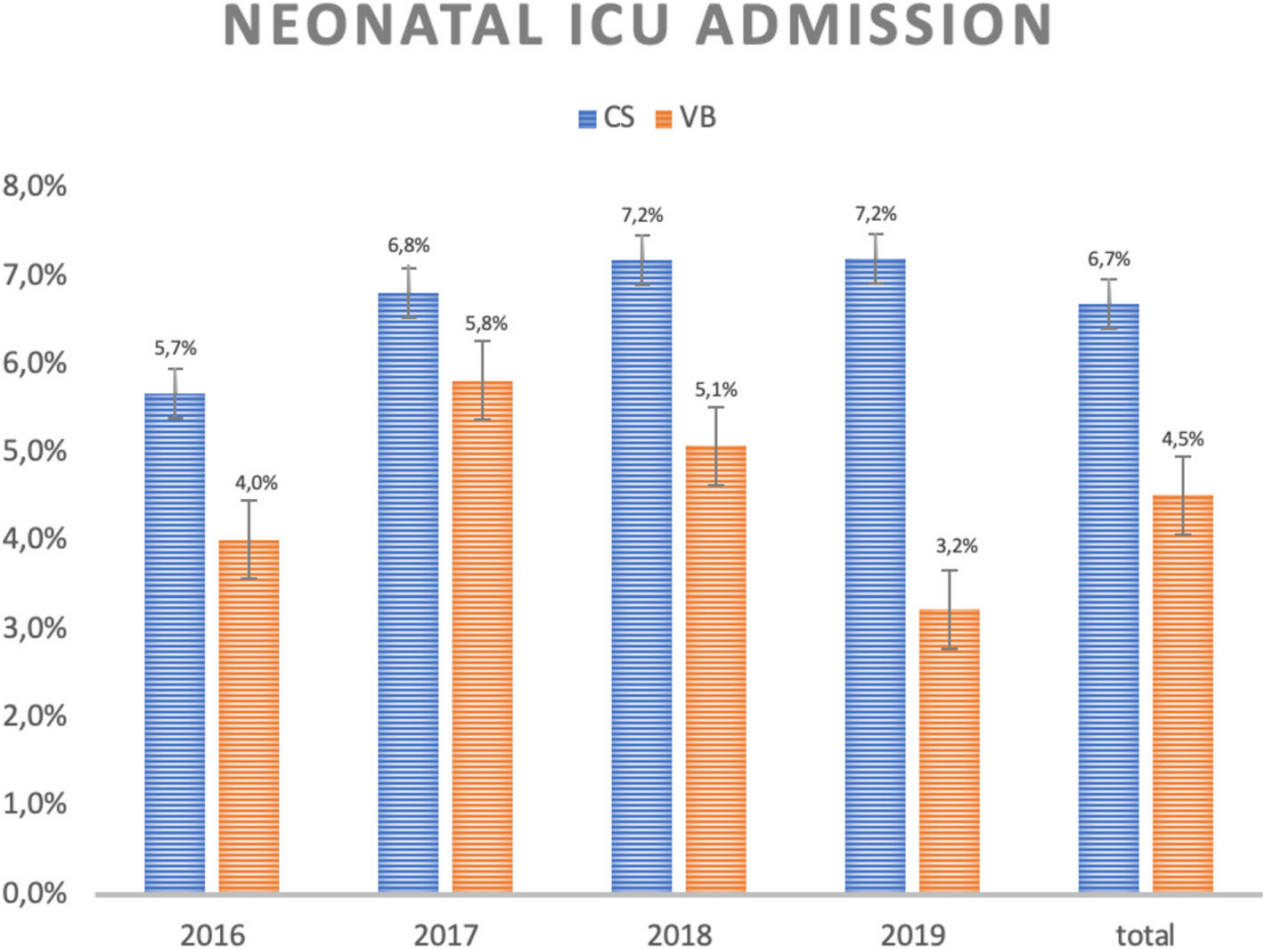
Neonatal ICU admission rate according to type of delivery and year of birth. CS = cesarean sections, VB = vaginal birth

Regarding the NICU admission rate, it is noticed that neonates born by C-section are more likely to need this type of support than those born by vaginal delivery (6.7% vs 4.5%, *p* = 0.0078, as shown in Fig. 2).

The quantitative analysis of NICU admissions in C-sections also reveals that in less than 5% of cases the cesarean was due to an emergency related to intrapartum fetal distress, so this condition seems not to contribute to the final result.

**Fig. 3:**
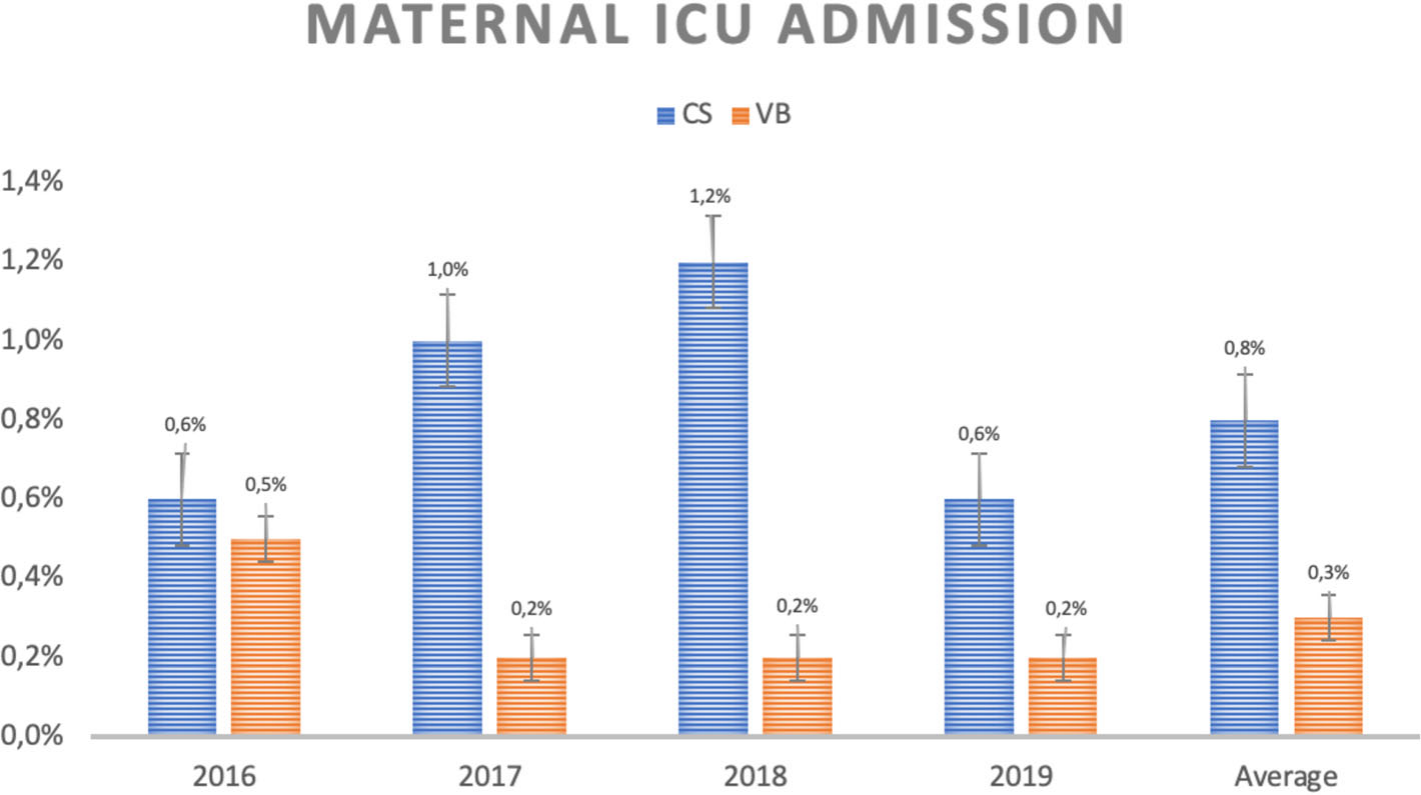
Maternal ICU admission rate according to type of delivery and year of occurrence. CS = C-sections, VB = vaginal birth

As with their babies, low-risk parturient who underwent C-sections had higher admission rates to the ICU than those who underwent vaginal delivery (0,8% vs 0,3%, *p* = 0.001), as shown in Fig. 3.

**Fig. 4:**
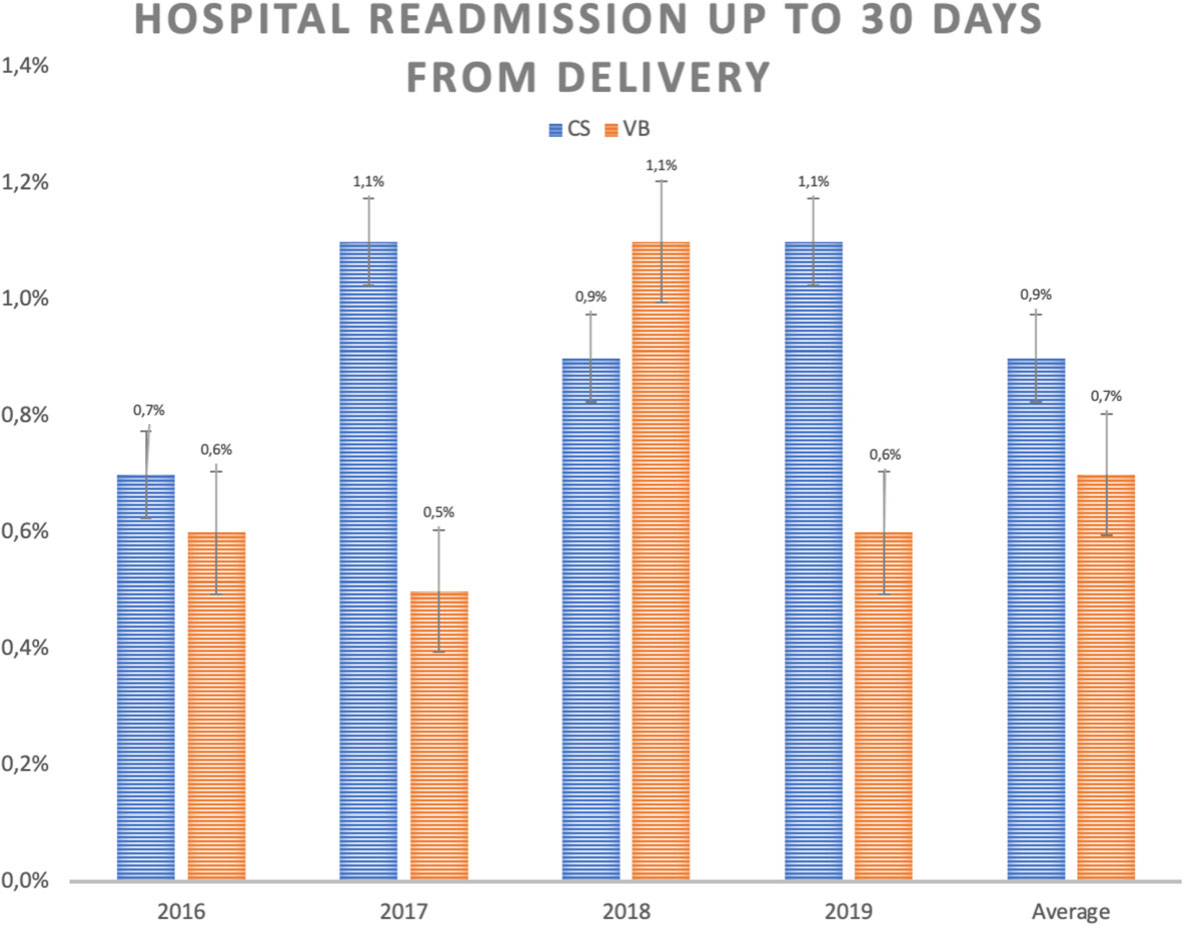
Hospital readmission rate up to 30 days from delivery according to type of delivery and year of occurrence. CS = C-sections, VB = vaginal birth

The rates of hospital readmission within 30 days from delivery are also higher in those patients submitted to C-sections than to vaginal delivery, although without statistical significance (Fig. 4).

**Fig. 5:**
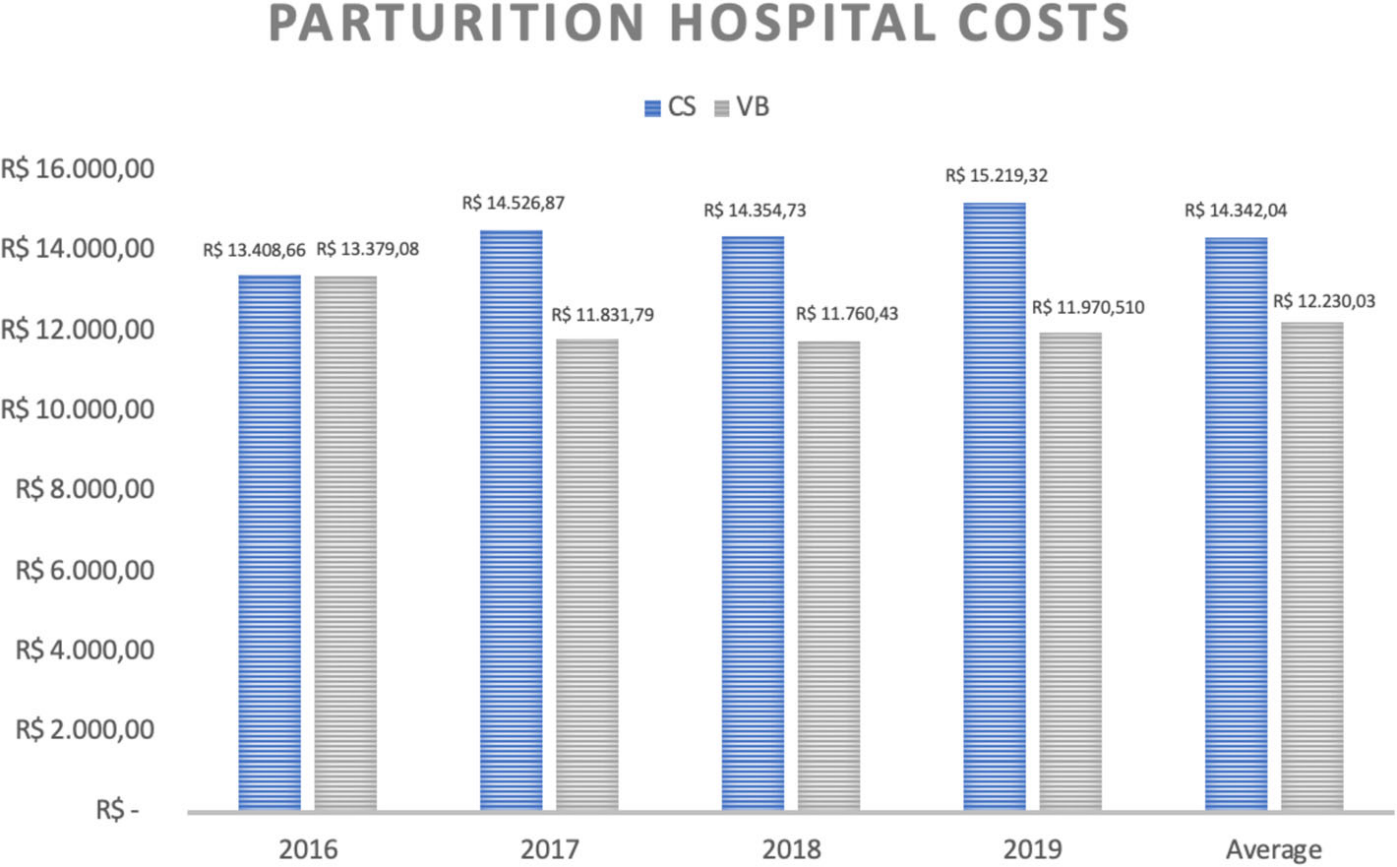
Average of parturition costs (in real) of hospitalization of the parturient-newborn binomial according to type of delivery and year of occurrence. CS = C-section, VB = Vaginal Birth

Finally, considering the average costs (calculated in Brazilian Reais – R$) of hospital stay for mother-baby binomial in low-risk pregnancies, it can be noted that cesarean deliveries cost R$14,0342.04 while vaginal deliveries cost R$12,230.03 (Fig. 5).

**Fig. 6:**
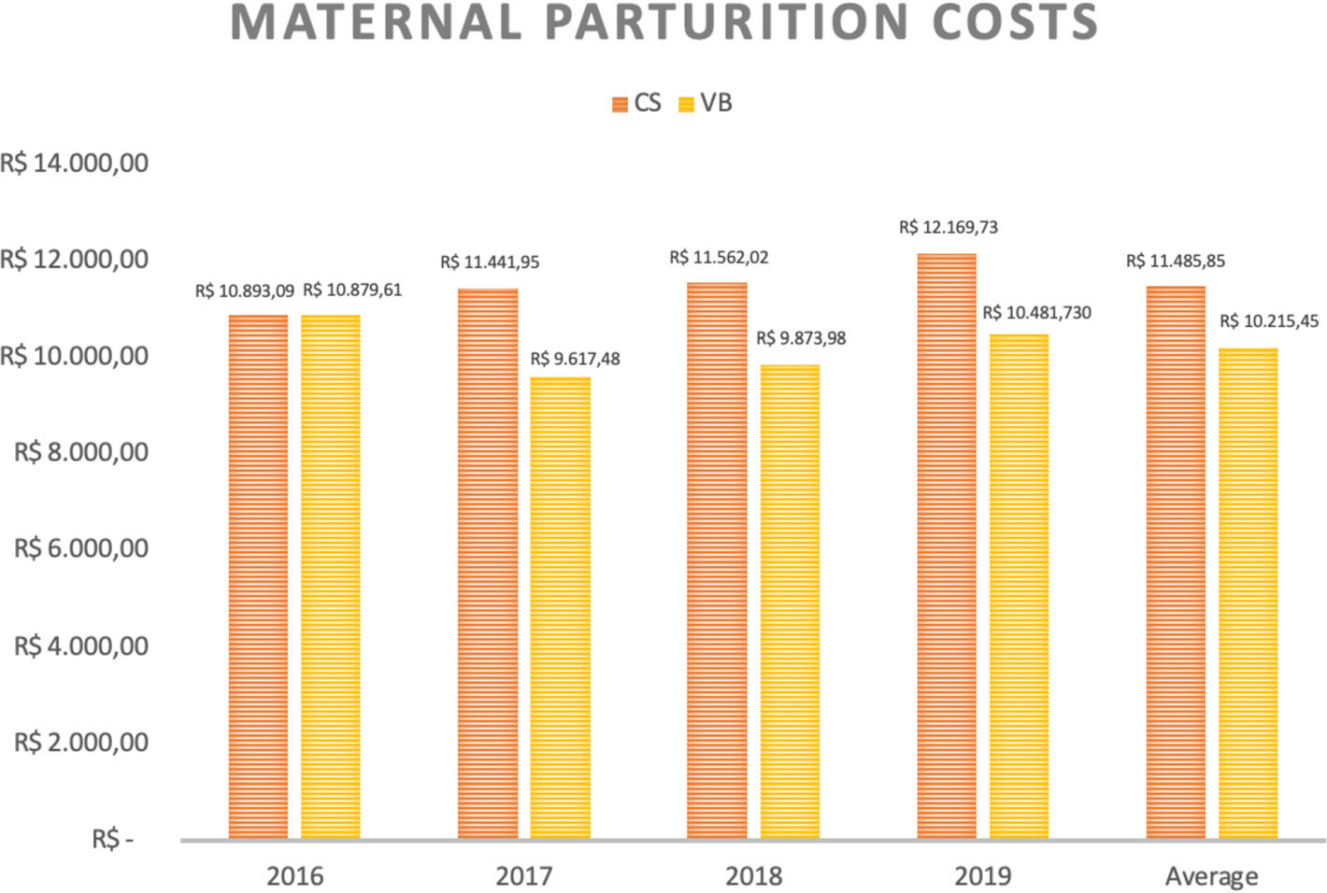
Average costs (in real) of maternal hospitalization for delivery according to type of delivery and year of occurrence. CS = C-section, VB = Vaginal birth

**Fig. 7:**
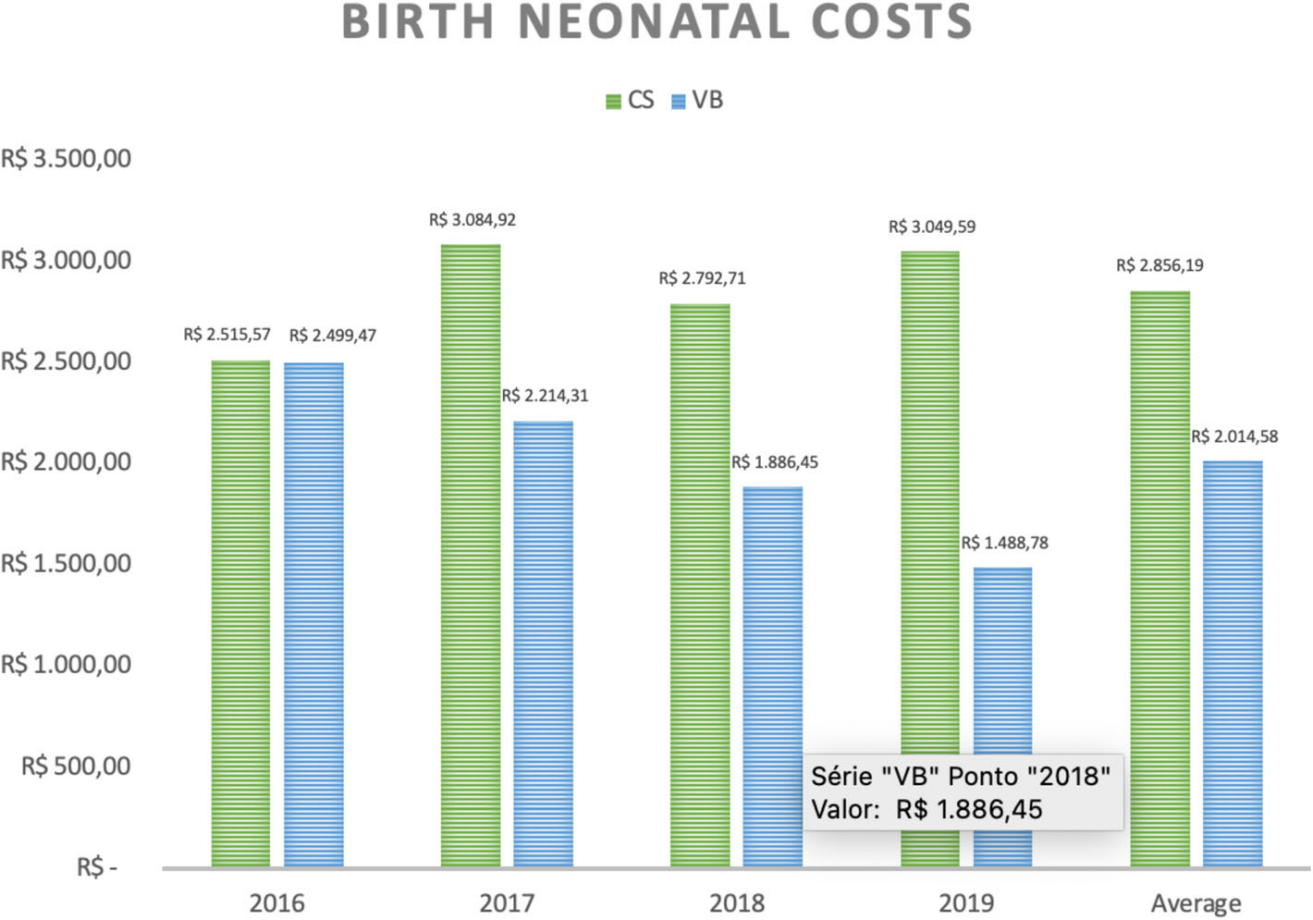
Average costs (in real) of hospitalization of the newborn due to delivery according to type of delivery and year of occurrence. CS = C-sections, VB = Vaginal birth

When analyzing maternal and neonatal hospital costs separately, we observed that C-sections present higher expenses for both settings, as demonstrated in Figs. 6 and 7.

## Discussion

Vaginal births have lower hospital costs than cesarean sections in low-risk pregnancies. Although C-section has been related to worse results [11], this study shows which kind of results are better in vaginal delivery, and includes only low risk pregnancies, in which C-sections should be less frequent.

The mothers’ results are better in vaginal birth considering ICU admission rates. The neonatal results are also more favorable in vaginal birth especially considering NICU admission and breastfeeding in the first hour after delivery. This study showed that vaginal births are associated with better healthcare value delivery.

Despite the absence of statistical significance, the maternal rates of hospital readmission within 30 days from delivery were higher in those patients undergoing C-sections than vaginal deliveries. This finding does not allow us to conclude that this is a worse clinical outcome, but it is still contributing for the analysis of medical expenses with C-sections.

Data analysis shows homogeneity between groups and this allows more substantial conclusions. The first item that draws attention is the comparison between the costs of cesarean and vaginal deliveries. Considering the overall hospital costs per patient (mother-baby binomial), cesarean deliveries are almost 15% more expensive than vaginal deliveries in low-risk pregnancies (R$14,0342.04 versus R$12,230.03). As the results showed, the C-sections higher costs must be related to the higher rate of maternal and neonatal ICU admission. Besides that, they may be related to the team needed to provide assistance for a C-section and increased use of medications in this kind of delivery.

Considering a scenario where C-section rates would represent 20% of cases for low risk pregnancies, and therefore still exceeding the 15–18% described as ideal and safe in the literature, the savings in the analyzed period would reach almost R$7million [7].

To imagine that such a rate is unattainable shows pessimism since the group studied was comprised exclusively by singleton pregnancies of low risk, with cephalic presentation and without previous C-section. In the available literature, low and high-risk pregnancies appear in the same universe of analysis. However, it is precisely the elective C-sections in low-risk groups that seem to promote the highest rate of preventable hospitalization in the NICU. Considering low risk as singleton pregnancies at term with cephalic presentation without previous C-section (Robson groups 1 to 4), which represents around 80% of births in the world, probably the action on these groups should translate in value results: lower costs and better outcomes [27].

There are already several attempts to reduce the number of caesareans worldwide, especially in Brazil, where these rates are incredibly high [28, 29].

The total cost of hospitalization for cesarean delivery is more expensive due to higher maternal and neonatal costs. Literature has shown that newborns of elective cesareans are more likely to have respiratory distress compared with those whose mothers went through labor [30]. Considering that such discomfort is the main cause of hospitalization in the NICU, greater use of this expensive resource in babies born by C- section was already expected [31]. Therefore, it is not surprising that there is a difference of almost 30% between hospital costs related to newborns undergoing cesarean delivery and those of vaginal delivery. Besides that, while only 4.5% of the babies born by vaginal delivery were admitted to NICU in this series, more than 6.7% of those born by C-section had the same outcome, which means a difference of approximately 160 admissions in this unit during the analyzed period. It is important to point out that intrapartum emergency C-sections only took place in exceptional conditions, such as fetal distress diagnosed by intrapartum cardiotocography category III and placental abruption.

From the maternal point of view, the results are also unfavorable for C-sections. The analysis of hospital readmission rate in the first 30 days after birth is one of the indicators proposed by the Health Care Payment Learning & Action Network working group. It reveals that it is more than 20% higher in cases of cesarean delivery than in vaginal delivery for the studied groups, although without statistical significance [26]. Maternal ICU admission rate also denotes a disadvantage for C-sections. Again, the causes are multifactorial but obviously closely related to the complications of the procedure. A retrospective study involving more than 14 thousand cases already exposed a higher rate of hospital readmission. These are mainly related to surgical wound infection in post-cesarean patients and that corroborates the data found in this series [32].

As proposed by the CQMC (Core Quality Measures Collaborative), breastfeeding in the first hour of life was considered as a value delivery [25]. Once again, the difference is in favor of vaginal delivery with an average adherence of 92% of cases versus 88% in C-sections. Factors such as postpartum position, anesthetic condition and surgical environment must have contributed to these results [33, 34] .

The remaining question is related to the continuity of a potentially harmful practice in relation to the other whose value delivery is shown to be greater. The answer to these questions is complex and involves several factors that generated the preference for C-section. They range from the model of remuneration for childbirth care to the judicialization of health, patient’s apprehension about a painful process, reduction of training in vaginal deliveries, cultural issues (such as fear of a painful process), and lack of active nursing support during labor [14].

Evidently, particularities about the assistance model of each country must be taken into account. In countries such as Brazil, where private assistance to labor is personalized, the assistance by a team in a shift and dichotomization between prenatal care and delivery may be plausible solutions when requiring more predictable availability from the assistance team. In other countries where this practice is already in vogue, the payment of bonus linked to outcomes could also bring benefits. Furthermore, cultural issues in countries that perceive C-section as the safer mode of delivery may also be addressed in educational campaigns.

Based on the above, it becomes clear that changes in the current form of obstetric care adapted to each economic model are urgent. Adjustments in the remuneration model may be important in countries with personalized and doctor-centered obstetric care. It is difficult to establish the ideal model, especially considering the differences between public and private systems.

However, ideas such as the use of the Global Budget defined by the average diagnosis-related-groups (DRG) of the target population and the payment of salaries to doctors have been used to manage hospitals. In this case, the hospital receives a fixed amount (monthly or yearly) to offer its services, regardless of the volume of resources it uses. With some nuances, as goals to be met and additional compensation by special procedures or accreditations, this model is used in some Social Health Organizations (OSS) in Brazil, in which non-profit companies manage government hospitals by fixed monthly values [35]. Also, a better structure of a bonus policy for professionals related to value delivery, besides the payment of salaries, could also be considered.

Finally, it is also necessary to build a better health system and medical teaching process to allow improvement in vaginal delivery rate, and consequently achieve better results and low costs regarding the delivery.

### Strengths and limitations

The strengths of this study are the detailed analysis of the causes for higher costs of cesarean sections and the description of worse results related to it. One clear advantage was the exclusion of high-risk cases that could cause an interpretation bias.

This study included a specific population and considered only low-risk pregnancies from a Brazilian private hospital. For that reason, it is essential to understand the reality of other medical institutions, both in clinical and cost-related terms. This is because vaginal delivery assistance can vary substantially in quality.

Besides that, the allocation of fixed and variable costs between different institutions may also differ. It is also necessary to understand whether the data can be replicated when the high-risk pregnancies are considered.

Finally, it could be stated that it is common to perform elective cesarean sections due to the maternal desire in the analyzed hospital, a reality that is not always present in every service and that could affect our results.

Data from a national hospital-based cohort with 23,940 postpartum women, held in 2011–2012, showed an initial preference for cesarean delivery of 27.6%, ranging from 15.4% (primiparous public sector) to 73.2% (multiparous women with previous cesarean private sector). The main reason for the choice of vaginal delivery was the best recovery of this type of delivery (68.5%) and for the choice of cesarean, the fear of pain (46.6%). Women from private sector presented 87.5% caesarean, with increased decision for cesarean birth in end of gestation, independent of diagnosis of complications. In both sectors, the proportion of caesarean section was much higher than desired by women [36].

## Conclusion

Cesarean deliveries in low-risk pregnancies were associated with a lower value delivery because, in addition to being more expensive, they had worse perinatal outcomes. Reviewing the financing model as well as the practice itself is essential to deliver more value-based healthcare in obstetrics.

## Data Availability

The data of this article are available with the corresponding author, by email romulo.negrini@einstein.br.

## References

[1] Xu K, Soucat A, Kutzin J (2018). Public spending on health: a closer look at global trends.

[2] Silveira D. 2019. Available at https://g1.globo.com/economia/noticia/2019/12/20/gasto-de-brasileiros-com-saude-privada-em-relacao-ao-pib-e-mais-que-dobro-da-media-dos-paises-da-ocde-diz-ibge.ghtml; Accessed Dec 2019.

[3] Barros A in IBGE News. 2019. Available at https://agenciadenoticias.ibge.gov.br/agencia-noticias/2012-agencia-de-noticias/noticias/26444-despesas-com-saude-ficam-em-9-2-do-pib-e-somam-r-608-3-bilhoes-em-2017; Accessed Dec 2019.

[4] Ribeiro A (2019). Observatório 2019.

[5] Médici A, Ribeiro A (2020). Observatório 2020.

[6] Agency for Healthcare Research and Quality (2011). HCUP Facts and figures: statistics on hospital based care in the United States, 2009.

[7] Healthcare Cost and Utilization Project (2008). Statistical brief: cost of childbirth.

[8] Agency for Healthcare Research and Quality (2003). HCUPNet, healthcare cost & utilization project. complications of pregnancy, childbirth, and the puerperium.

[9] Agency for Healthcare Research and Quality (2013). HCUPNet, healthcare cost & utilization project. certain conditions originating in the perinatal period.

[10] Global Date (2015). Forthcoming report about preterm births.

[11] Caughey AB, Cahill AG, Guise JM, Rouse DJ, American College of Obstetricians and Gynecologists (College); Society for Maternal-Fetal Medicine (2014). Safe prevention of the primary cesarean delivery. Am J Obstet Gynecol.

[12] Leite AC, Araujo Júnior E, Helfer TM, Marcolino LA, Vasques FA, Sá RA (2016). Comparative analysis of perinatal outcomes among different typesof deliveries in term pregnancies in a reference maternity of Southeast Brazil. Ceska Gynekol.

[13] Etringer AP, Pinto MFT, Gomes MASM. Análise de custos da atenção hospitalar ao parto vaginal e à cesariana eletiva para gestantes de risco habitual no Sistema Único de Saúde. Cien Saude Colet. 2019;24(4). 10.1590/1413-81232018244.06962017 31066854

[14] Truven Health Analytics (2013). The cost of having a baby in the United States.

[15] Molina G, Weiser TG, Stuart R (2015). Relationship between cesarean delivery rate and maternal and neonatal mortality. JAMA.

[16] SINASC/DATASUS. Available at (last accessed in April, 2020): http://www2.datasus.gov.br/DATASUS/index.php?area=060702.

[17] Vogt SE, Silva KS, Dias MAB (2014). Comparison of childbirth care models in public hospitals, Brazil. Rev Saude Publica.

[18] Brown MM, Brown GC, Brown HC, Irwin B, Brown KS (2008). The comparative effectiveness and cost-effectiveness of vitreoretinal interventions. Curr Opin Ophthalmol.

[19] Bae J (2015). Value-based medicine. Epidemiol Health.

[20] Porter ME (2010). What is value in health care?. N Engl J Med.

[21] Institute of Medicine (2001). Crossing the quality chasm: a new health system for the 21st century.

[22] Institute for Healthcare Improvement (2016). QCV100: an introduction to quality, cost, and value in health care.

[23] Quinn K (2015). The 8 basic payment methods in health care. Ann Intern Med.

[24] OCDE Focus on. Better ways to pay for health care. Available at (last accessed in Apr 2020): https://www.oecd.org/els/health-systems/Better-ways-to-pay-for-health-care-FOCUS.pdf. Jun 201.

[25] U.S. Department of Health and Human Services, Centers for Medicare & Medicaid Services (2016). Core measures.

[26] Healthcare Payment Learning and Action Network (2018). Accelerating and aligning clinical episode payment models.

[27] Vogel JP, Betrán AP, Vindevoghel N, Souza JP, Torloni MR, Zhang J, et al. Use of the Robson classification to assess caesarean section trends in 21 countries: a secondary analysis of two WHO multicountry surveys. Lancet Glob Health. 2015;3(5):e260–70. 10.1016/S2214-109X(15)70094-X. 10.1016/S2214-109X(15)70094-X 25866355

[28] Negrini R, Ferreira RDDS, Albino RS, Daltro CAT. Reducing caesarean rates in a public maternity hospital by implementing a plan of action: a quality improvement report. BMJ Open Qual. 2020;9(2). 10.1136/bmjoq-2019-000791 PMC7223294 32381595

[29] Borem P, de Cássia SR, Torres J (2020). A quality improvement initiative to increase the frequency of vaginal delivery in Brazilian hospitals. Obstet Gynecol.

[30] Curet LB, Zachman RD, Rao AV, Poole WK, Morrison J, Burkett G (1988). Effect of mode of delivery on incidence of respiratory distress syndrome. Int J Gynecol Obstet.

[31] Yee W, Amin H, Wood S (2008). Elective cesarean delivery, neonatal intensive care unit admission, and neonatal respiratory distress. Obstet Gynecol.

[32] Ergen EB, Ozkaya E, Eser A (2018). Comparison of readmission rates between groups with early versus late discharge after vaginal or cesarean delivery: a retrospective analyzes of 14,460 cases. J Matern Fetal Neonatal Med.

[33] Brown A, Jordan S (2013). Impact of birth complications on breastfeeding duration: an internet survey. J Adv Nurs.

[34] Zanardo V, Svegliado G, Cavallin F, Giustardi A, Cosmi E, Litta P, et al. Elective cesarean delivery: does it have a negative effect on breastfeeding? Birth. 2010;37(4):275–9. 10.1111/j.1523-536X.2010.00421.x PMID: 21083718. 10.1111/j.1523-536X.2010.00421.x 21083718

[35] Morais HMM, Albuquerque MSV, Oliveira RS, Cazuzu AKI, Silva NAFD (2018). Social healthcare organizations: a phenomenological expression of healthcare privatization in Brazil. Cad Saúde Pública.

[36] Domingues RMSM, Dias MAB, Nakamura-Pereira M (2014). Process of decision-making regarding the mode of birth in Brazil: from the initial preference of women to the final mode of birth. Cad Saúde Pública.

